# A Transcriptome Study on Seed Germination of *Nitraria roborowskii* Kom.

**DOI:** 10.3390/ijms27031442

**Published:** 2026-01-31

**Authors:** Shangfu Ren, Guanghui Lv

**Affiliations:** 1 College of Life and Geographic Sciences, Kashi University, Kashi 844006, China; 2 Key Laboratory of Biological Resources and Ecology of Pamirs Plateau in Xinjiang Uygur Autonomous Region, Kashi 844006, China; 3 College of Ecology and Environment, Xinjiang University, Urumqi 830017, China; guanghui_xju@sina.com; 4 Key Laboratory of Oasis Ecology of Education Ministry, Xinjiang University, Urumqi 830017, China; 5 Xinjiang Jinghe Observation and Research Station of Temperate Desert Ecosystem, Ministry of Education, Jinghe 833399, China

**Keywords:** *Nitraria roborowskii* Kom., seeds, dormancy release, genes, differential expression

## Abstract

*Nitraria roborowskii* Kom. seeds possess pronounced deep dormancy traits. Analyzing changes in gene expression before and after dormancy release is of great significance for elucidating the mechanisms underlying seed dormancy. In this study, transcriptome sequencing and bioinformatics analysis were conducted on *N. roborowskii* seeds both before and after dormancy release using high-throughput Illumina NovaSeq 6000 sequencing technology. The key findings are as follows: (1) A total of 215,303 transcripts and 84,450 unigenes were obtained through de novo assembly. (2) Comparative analysis revealed 16,130 significantly differentially expressed unigenes during germination, with 10,776 upregulated and 5354 downregulated. Gene Ontology (GO) enrichment analysis indicated that these differentially expressed genes (DEGs) were primarily associated with biological processes and molecular functions, mainly involved in metabolic processes and catalytic activities. (3) Kyoto Encyclopedia of Genes and Genomes (KEGG) pathway enrichment analysis showed that the DEGs were predominantly enriched in pathways such as plant hormone signal transduction and starch and sucrose metabolism. Specifically, among the downregulated genes, 126 were linked to plant hormone signal transduction, 110 to phenylpropanoid biosynthesis, 108 to starch and sucrose metabolism, 27 to flavonoid biosynthesis, 20 to plant hormone signal transduction, 6 to phenylpropanoid metabolism, 14 to starch and sucrose metabolism, and none to flavonoid biosynthesis.

## 1. Introduction

As an optimal plant for improving saline–alkali conditions, *Nitraria roborowskii* Kom. belongs to the genus Nitraria of the Nitrariaceae [1,2], and its seeds exhibit deep dormancy characteristics [3,4,5]. *N. roborowskii* has not only important ecological value but also nutritional value, medicinal value and economic value. Seeds of the Nitraria genus reportedly exhibit physiological dormancy (PD) [6]. The physiological dormancy of seeds is a double inhibition mechanism due to reduced embryo vigor coupled with the restriction of gas exchange caused by the seed coat. Short-term cold stratification, light, dry storage, disruption of the outer coat, and growth promotion can disrupt the physiological dormancy of seeds [7]. According to PIM intensity, physiological dormancy can be divided into three categories: shallow (C1), moderate (C2), and strong (C3) physiological dormancy. PD is the most abundant form and is found in the seeds of gymnosperms and in all major angiosperm clades [8].

The transition of seeds from the dormant stage to the germination stage involves very complex biological processes [8]. There are often more differentially expressed genes (DEGs) between the two phases [9]. Therefore, exploring the dormancy mechanism of *N. roborowskii* seeds is valuable for their development and utilization. Transcriptome research has become an effective means for discovering key genes involved in seed germination [10]. It can comprehensively reveal differences in gene expression under different physiological conditions and has been widely used for screening genes related to the low-temperature stress response and participating in the seed germination process [11]. In recent years, differential gene studies related to seed dormancy enhancement have been carried out on *Polygonatum sibiricum* Red [12] and *Paris polyphylla* [13].

The lack of *Nitraria sibirica* Pall. Information on its genome limits further exploration of the molecular mechanisms involved in salt stress [14]. To date, no transcriptome studies have been conducted on the dormancy release of large white thorn fruit seeds. Therefore, in this study, transcriptome sequencing was performed on samples before and after dormancy release from *N. roborowskii* seeds to address the following scientific questions: (1) Which key genes were significantly differentially expressed after dormancy release from *N. roborowskii* seeds? (2) Which metabolic pathways underwent critical changes?

## 2. Results

### 2.1. Transcriptome Sequencing and De Novo Assembly

Since there is no corresponding reference gene sequence for *N. roborowskii*, this study used the de novo assembly method and Trinity software to assemble the clean reads. More than 20,529,105 raw reads were obtained for each sample, and after filtration, more than 5.8 Gb of sequencing data were obtained. Q20 ≥ 97.20%, Q30 ≥ 91.80%, and the GC content ranged from 44.69~50.19% (Table 1). A total of 215,303 transcripts and 84,450 unigenes were obtained, with the maximum lengths of the transcripts and unigenes being 13,381 bp and 1614 bp, respectively, and the N50 length was 1461 bp. A total of 84,450 unigenes were annotated in the NT, NR, KOG/COG, Swiss-Prot, PFAM, KEGG, and GO databases, and the annotation rates of the unigenes in each database are shown in Table 2.

### 2.2. Expression Levels of Genes Before and After Germination

The percentages of unigenes with an FPKM [15] > 0.3 were 34.36%, 28.41%, 38.79%, 82.37%, 51.96% and 53.34%, respectively, indicating that the gene expression level of the seeds of *N. roborowskii* in the dormant period was relatively low, with an average value of 33.85%, of which A3 was the highest at 38.79%, and after the seeds of *N. roborowskii* were lifted during the dormant period, their gene expression level increased greatly, with an average value of 62.56%, of which B1 was the highest at 82.37%.

**Figure 1:**
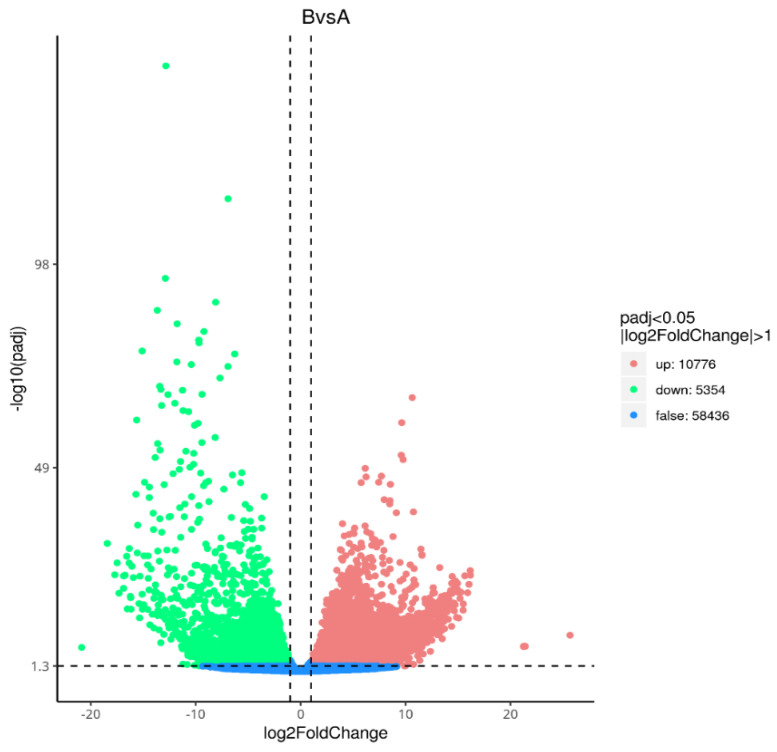
Volcano map of differentially expressed genes.

According to the experimental sequencing data, the transcriptomes of dormant seeds (A) and dormant-released seeds (B) of *N. roborowskii* were compared and analyzed, and a total of 16,130 DEGs were identified, of which 10,776 genes were upregulated and 5354 genes were downregulated (Figure 1).

Note: The green dots represent significantly downregulated genes, the red dots represent significantly upregulated genes, and the blue dots represent genes whose expression did not significantly change. The X-axis indicates the base-2 logarithms of the fold changes of the DEGs, and the Y-axis indicates the negative base-10 logarithms of the *p* values of the DEGs.

### 2.3. Analysis of Gene Function Annotation Differences Before and After Germination

**Figure 2:**
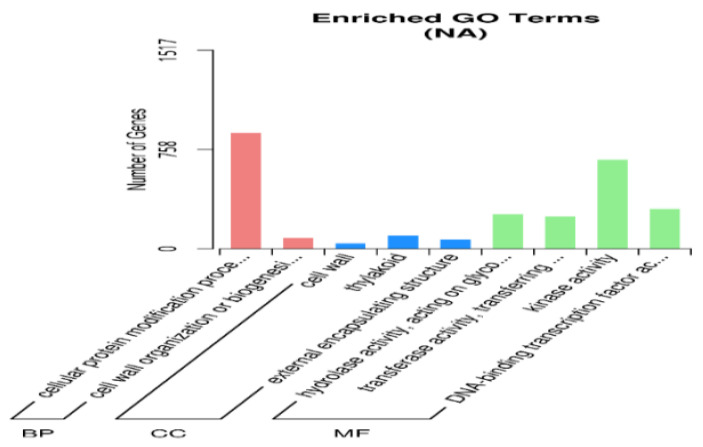
Differential Gene GO Enrichment Column Diagram.

The DEGs were annotated into 3 categories [16], BP (16,549), CC (10,583), and MF (10,377), accounting for 44.12%, 28.21%, and 27.67%, respectively. Among them, 6639 differentially expressed genes in BP were annotated to 66 functions (Figure 2), including mainly cellular nitrogen compound metabolic processes, biosynthetic processes, and transport. There were 4141 DEGs in CC, annotated to 32 functions, and the 3 most important functions were intracellular, protein-containing complex, and organelle. There were 7528 DEGs in the MF category, which were annotated to 37 functions, and the most annotated genes were involved in ion binding, oxidoreductase activity, and DNA binding. The functions of these genes are related to metabolic activities.

**Figure 3:**
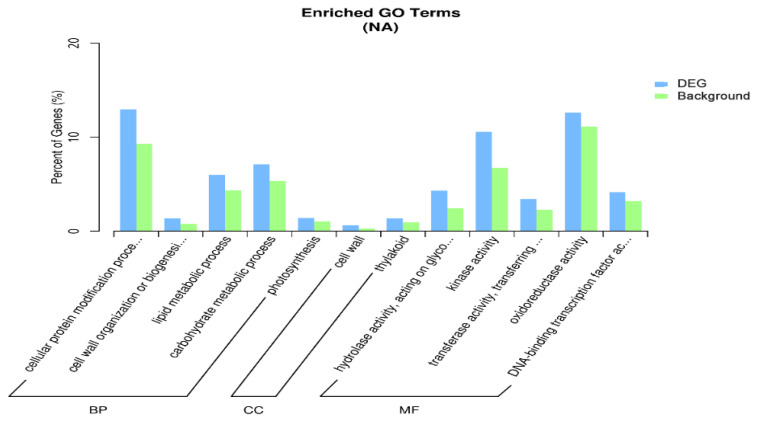
GO enrichment analysis of the upregulated genes.

**Figure 4:**
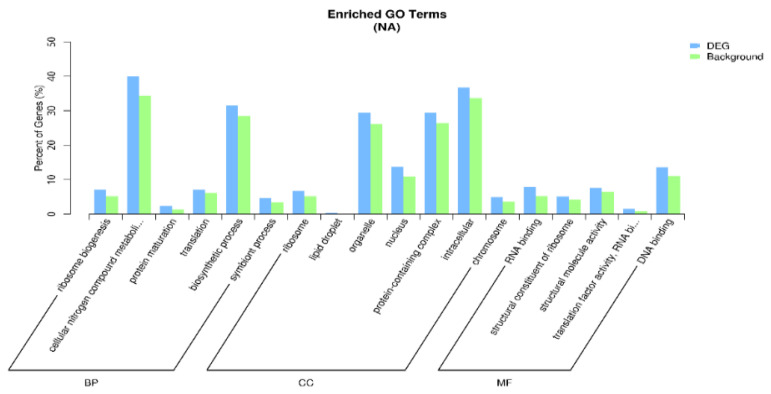
GO enrichment analysis of downregulated genes.

During the process of seed dormancy release, the 3 most significantly upregulated annotated genes in the 3 most important biological processes were “cellular protein modification processes”, “cell wall organization or biogenesis”, and “lipid metabolic processes”; the 3 most important cellular components were “cell wall”, “thylakoid”, and “external encapsulation structure”; and the 3 most important molecular functions of the annotated genes were “hydrolase activity acting on glycosyl bonds”, “kinase activity”, and “transferase activity of glycosyl groups” (Figure 3). The 3 most important biological processes with significantly downregulated annotated genes were ribosome biogenesis, the cellular nitrogen compound metabolic process, and mature protein; the 3 most important cellular components were ribosome, lipid droplet, and organelle; and the 3 most important molecular functions were RNA binding protein, ribosome structural constituent, and structural molecule activity (Figure 4).

### 2.4. Analysis of Gene Expression Differences Before and After Germination

**Figure 5:**
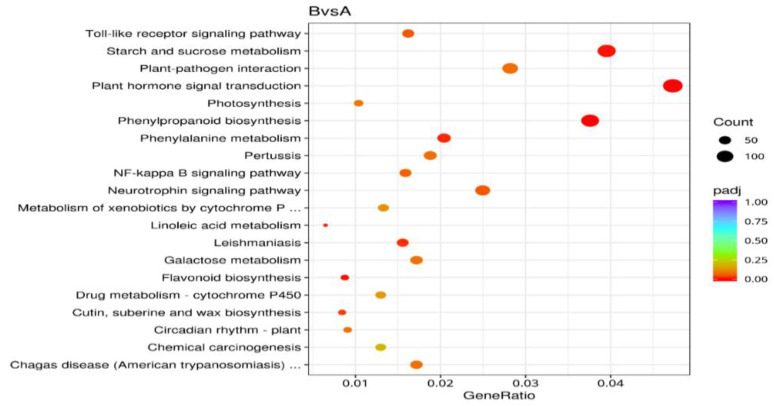
Statistics of pathway enrichment of DEGs.

Note: The X-axis represents the enrichment factors of different KEGG pathways, and the Y-axis indicates different KEGG pathways. The different colors of the dots indicate different q-values. The different sizes of the dots indicate different numbers of genes.

**Figure 6:**
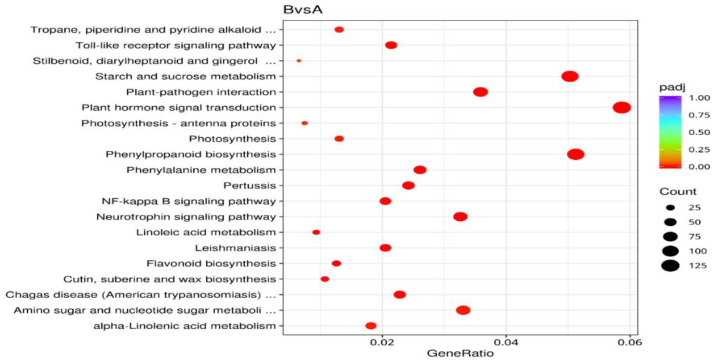
Statistics of pathway enrichment of upregulated genes.

**Figure 7:**
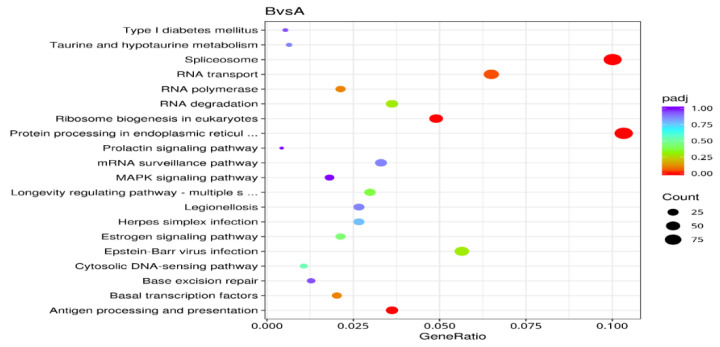
Statistics of pathway enrichment of downregulated genes.

The top 20 pathways with the largest number of genes were included [17] (Figure 5). The five pathways with the greatest enrichment were plant hormone signal transduction, starch and sucrose metabolism, phenylpropanoid metabolism, plant–pathogen interaction, and neurotrophic protein signaling pathways. For the upregulated genes, the top 20 pathways with the most significant enrichment were selected (Figure 6), and the five pathways with the greatest enrichment were plant hormone signal transduction, phenylpropanoid metabolism, starch and sucrose metabolism, plant–pathogen interaction, and the neurotrophic protein signaling pathway. For the downregulated genes, the top 20 pathways with the most significant enrichment were selected (Figure 7), and the five pathways with the greatest enrichment were splicing, protein processing in the endoplasmic reticulum, RNA transport, Epstein–Barr virus infection, and ribosome biosynthesis in eukaryotes. In this study, a total of 10 metabolic pathways related to the release of dormancy in *N. roborowskii* were identified (Table 3).

### 2.5. Differentially Expressed Genes in the Hormone Signal Transduction Pathway Before and After Germination

Studies have shown that genes involved in plant hormone signal transduction play a key role in the process of seed dormancy and germination. The DEGs of *N. roborowskii* seeds before and after germination were associated with the plant hormone signal transduction pathway (ko04075). The Q value also revealed that the hormone signal transduction pathway presented the greatest difference in gene expression. The results revealed 146 annotated DEGs in the hormone signal transduction pathway (Table 4), of which 126 genes were upregulated and 20 genes were downregulated. The numbers of genes associated with GA, ABA, IAA, CTK, ETH, BR and JA were 15, 5, 4, 3, 3, 6, 7 and 5, respectively (Table 5).

## 3. Discussion

Seed dormancy is regulated by a variety of genes, and genes encoding phytohormones and key enzymes of the respiratory pathway play important roles in the regulation of seed dormancy release. A total of 44 genes encoding three key enzymes, namely, phosphoglucose isomerase (PGI), which is involved in glycolysis (EMP); malate dehydrogenase (MDH), which is involved in the tricarboxylic acid cycle (TCA); and glucose-6-phosphate dehydrogenase (G6PDH), which is involved in the pentose phosphate pathway (PPP), were found in the transcriptomes of *N. roborowskii* seeds. Among the dormancy lifting and dormant *N. roborowskii* seeds with key respiratory pathway metabolism enzyme-related DEGs, 11 DEGs were upregulated, while a total of 8 *PGI* genes and 7 genes were upregulated. A total of 31 *MDH* genes and 26 genes were upregulated, and a total of 5 *G6PDH* genes were upregulated; i.e., three key enzymes were upregulated, and the three key enzymes were significantly upregulated in the process of increasing seed dormancy in *N. roborowskii.* High α-amylase gene expression is an important indicator of increased seed dormancy. In this study, we determined that there were 11 differentially expressed α-amylase genes in the transcriptome of the seeds of *N. roborowskii*, of which 8 were significantly upregulated and 3 were significantly downregulated. The elevated content of α-amylase promoted the conversion of starch to soluble sugars in the seeds, and the high and low contents were related to the low-temperature resistance of the plants, which is in line with the preliminary findings. This finding is consistent with the conclusion of the study that the soluble sugar content increased after the increase in seed dormancy in *N. roborowskii*.

In this study, GO enrichment analysis of DEGs in *N. roborowskii* seeds before and after germination revealed that the DEGs were involved mainly in metabolic processes, catalytic activity, single-organism metabolic processes, oxidoreductase activity, and ribosomes. KEGG and weighted gene co-expression network analyses indicated that DEGs responsive to saline-alkaline stress were predominantly enriched in pathways including plant hormone signal transduction, MAPK signaling, starch and sucrose metabolism, glutathione metabolism, and phenylpropanoid biosynthesis [18]. KEGG pathway enrichment analysis revealed that the genes that were significantly differentially expressed after seed germination were enriched mainly in pathways such as ribosome, carbon metabolism, amino acid biosynthesis, and protein processing. Among them, 146 genes were involved in the hormone signal transduction pathway, 116 genes were involved in the phenylpropanoid metabolism pathway, 27 genes were involved in the flavonoid biosynthesis pathway, 122 genes were involved in the starch and sucrose metabolism pathways, and many DEGs were significantly expressed in the MAPK signaling pathway, which is consistent with previous studies.

The dormancy of Ginkgo biloba seeds is regulated not only by the balance of ABA/GA but also by other hormones related to embryo morphological development and genes related to embryo differentiation and development [19]. KEGG pathway analysis revealed that plant hormone signal transduction is related to seed germination, and identifying hormone metabolic genes and analyzing gene expression patterns are the keys to understanding the regulation of dormancy and germination [7,20].

The GA signaling pathway consists mainly of target factors regulated by the receptor *GID1* [21]. *GID1* is a soluble GA receptor that is localized in the nucleus and cytoplasm of rice and *Arabidopsis* cells [22]. The expression of genes related to gibberellic acid (GA) catabolism and signal transduction (*CYP707As*, *GA2ox*, and *DELLAs*) was consistent with endogenous hormone changes [23]. This study compared the transcriptome data of dormant and dormant-release *N. roborowskii* seeds and identified a total of 80 genes related to plant hormone (ABA, GA, and IAA) metabolism, among which the GA receptor (*GID1A*) was significantly upregulated by 1.47-fold. After the dormancy of *N. roborowskii* seeds was released, the expression of the *SLY1* gene increased significantly. A *sleepy1* (*sly1*) mutant was identified in *Arabidopsis* seeds, and *SLY1* was hypothesized to be a key factor in seed sensory gibberellin signaling [24]. GA promotes the generation of growth potential by stimulating cell elongation and inducing the expression of genes encoding cell wall-modifying enzymes, such as the expansin gene (*EXP*), which can regulate cell wall loosening and is important for cell elongation in dormant-released *N. roborowskii* seeds. During the process of releasing the dormancy of *N. roborowskii* seeds, the expression of genes related to GA synthesis (*GA2OX1*, *GA2OX2*, *GA2OX6* and *GA2OX8*) and gibberellin regulatory proteins (*GASA4*, *GASA9*, *GASA13* and *GASA14*) increased significantly. Five *C19-GA2ox* genes have been identified in *Arabidopsis thaliana* [25].

The expression of the GA-positive regulatory gene *PP2A* significantly differed before and after the release of dormancy in *N. roborowskii* seeds. Several studies have shown that the molecular marker of the germination period phosphatase (*PP2A*), which is involved in the release of dormancy in *Arabidopsis* seeds, is upregulated in *GCR1* plants [26].

In this study, the expression of ABA biosynthetic 9-cis-epoxycarotenoid dioxygenase (*NCED6*), which is involved in the ABA metabolic signal transduction pathway, was significantly downregulated 8.14-fold. The *NCED6* gene is expressed specifically in seeds, plays a major role in ABA synthesis during seed development and germination and can induce seed dormancy [27]. Under low-temperature conditions, the expression of genes involved in ABA synthesis (*NCED*) decreases, whereas the expression of genes involved in gibberellin and ethylene synthesis (*GA3oxl* and *ACS1*) increases [28]. In this study, the expression of *NCED* decreased, and the expression of *ACS1* increased. The ABA content in seeds is related not only to the synthetic pathway but also to its inactivation. The *CYP707A2* gene encoding ABA-8′-hydroxylase is a key protein that regulates ABA metabolism. In this study, the expression of *CYP707A2*, which is a key regulatory enzyme involved in the release of dormancy in *N. roborowskii* seeds, was significantly upregulated by 4.13-fold. Studies have shown that the *DELLA* gene family is the main factor regulating GA signaling [29]. The expression of *DELLA* genes (*GAI* and *GAIP*) in *N. roborowskii* seeds was upregulated, which proved that the GA content increased and that ABA synthesis decreased or decreased in the seeds released from dormancy, which jointly promoted the germination of *N. roborowskii* seeds. The results of this experiment revealed that 40 *BHLH* genes in the seeds of *N. roborowskii* released from dormancy were significantly upregulated. *BHLH* transcription factors indirectly regulate the ABA synthesis gene *NCED2*, which can regulate ABA metabolism and synthesis and achieve accurate regulation of ABA in seeds, thereby affecting the process of seed dormancy or germination [30]. The relative expression levels of ABA synthesis- and metabolism-related genes change [31]. *ABI5* shares several target genes that negatively regulate embryo germination together with the *bHLH* transcription factor *PIF1* [32].

## 4. Materials and Methods

### 4.1. Experimental Materials

The seeds of *N. roborowskii* required for this experiment were purchased from the planting base of Minqin County Linquan Ecological Seed Industry Co., Ltd. (Wuwei, China). According to the seed collection requirements specified in the “Technical Code for Sowing and Seedling Cultivation of Nitraria (LY/T 2295-2014),” the following procedures were followed during fruit harvesting: healthy, tall, and high-yielding mother plants were selected. Fruits showing signs of pests or diseases were excluded. The fruits were manually picked or shaken from the branches and collected separately to prevent mixing. After natural air-drying, the dried *N. roborowskii* fruits were soaked in water until fully expanded. They were then placed in an iron sieve with a mesh size smaller than the seed diameter and repeatedly rubbed to break down the pulp. The mixture was rinsed under running water to remove floating peels, empty grains, rotten grains, and pest-damaged grains. After multiple rounds of rubbing and rinsing, clean seeds were obtained. These seeds were air-dried naturally, packaged in sealed bags, and stored in a refrigeration chamber.

### 4.2. Material Treatment

The seeds of *N. roborowskii* for this study were sourced from the Minqin County Linquan Ecological Seed Industry Co., Ltd. (Wuwei, China) planting base. Mature, healthy seeds (viability: 83.00% by TTC method) were selected, surface-sterilized with 0.1% KMnO_4_ for 30 min, and then soaked in 500 mg/L GA_3_ for 24 h, with distilled water as control. The GA_3_-treated seeds were mixed with moist river sand (1:3, mass ratio; ~75% moisture—assessed as “hand-squeezed into a ball, crumbles when released”) and subjected to outdoor sand stratification for 90 days. Radicle emergence was taken as the criterion for dormancy break. Most GA_3_-treated seeds showed radicle and plumule exposure, indicating dormancy release, whereas control seeds displayed no germination signs and remained dormant [33]. Seed embryos in dormant (A1, A2, A3) and dormancy-released (B1, B2, B3) states were separately used for RNA extraction and transcriptome sequencing. For each sample, approximately 0.1 g material was collected with three biological replicates.

### 4.3. Total RNA Extraction, Library Construction and Sequencing

RNA was extracted via the cetyltrimethylammonium bromide (CTAB) method (QIAGEN, Germany). The Panomics default sequencing platform used was an Illumina NovaSeq 6000 (Illumina, San Diego, CA, USA) with S4 suite components. After the library was constructed, it was first quantified via a Qubit 2.0 fluorometer, and then the insert size of the library was detected via an Agilent 2100 bioanalyzer. After the insert size met the expectations, the effective concentration of the library was accurately quantified via qRT–PCR (the effective concentration of the library was greater than 2 nM) to ensure the quality of the library.

### 4.4. Quality Control and Assembly

Since there is no reference genome for *N. roborowskii* seeds, Trinity [34] was used to assemble the clean reads to obtain the unigene library during the process of seed dormancy release. In this study, the de novo assembly method was used, and Trinity software (v2.4.0) was used to assemble the clean reads. With Corest software (v4.6), the transcripts were clustered and found to be redundant, and each cluster was ultimately defined as a unigene. KEGG annotations were performed on the unigenes via KAAS, and GO annotations were performed via Blast2 GO.

### 4.5. Gene Functional Annotation

To obtain the functional information of the sequence genes, all the unigenes after assembly were subjected to BLAST (v2.0.6) [35] searches against seven functional databases, namely, the NR, NT, PFAM, COG, Swiss-Prot, KEGG, and GO databases, via BLASTX (v2.0.6).

### 4.6. Data Analysis

The transcript sequences obtained in this study were used as reference sequences, and RSEM [36] was used to map the clean reads to the reference sequence with Bowtie2 via default parameters. Through a BLASTx search, the key genes involved in the dormancy and germination of *N. roborowskii* seeds were identified.

## 5. Conclusions

In this study, using *N. roborowskii* seeds before and after dormancy release as the research subjects, a total of 16,130 differentially expressed genes (DEGs) were identified, of which 10,776 were upregulated and 5354 were downregulated. Among these DEGs, 165,450 were functionally annotated by GO classification into three main categories and 43 subcategories, covering biological processes, molecular functions, and cellular components. In KEGG pathway analysis, 36,540 DEGs were mapped to 303 metabolic pathways, with notable involvement in pathways such as the ribosome, carbon metabolism, biosynthesis of amino acids, protein processing in the endoplasmic reticulum, and spliceosome. Based on the annotation results from the KEGG database, ten metabolic pathways closely related to the dormancy release of *N. roborowskii* seeds were identified. Among these, the plant hormone signal transduction pathway and the starch and sucrose metabolism pathway play key regulatory roles during seed germination. Additionally, this study identified key genes (*GID1A*, *GA2OX*, *PP2A*, *NCED*, *CYP707A2*, *SLY1*, *EXP*, *GASA*, and *BHLH*) that exhibited significant expression changes before and after seed germination.

## Figures and Tables

**Table 1:** Quality statistics of sequencing reads.

Sample	Raw Reads/Mb	Clean Reads/Mb	Clean Bases/Gb	Error Rate (%)	Q20 (%)	Q30 (%)	GC Content (%)
A1	2,2209,717	20,599,156	6.2	0.03	97.14	92.69	47.05
A2	20,529,105	20,418,702	6.1	0.03	96.52	91.80	44.69
A3	22,215,352	21,185,006	6.4	0.03	97.20	92.74	47.27
B1	21,403,752	19,948,928	6.0	0.03	97.37	92.97	50.19
B2	21,022,361	19,391,317	5.8	0.03	97.40	92.93	44.98
B3	22,306,221	21,229,151	6.4	0.03	97.37	92.86	45.13

**Table 2:** Unigene annotation rate.

Database	Number of Unigenes	Percentage (%)
Annotated in NR	52,557	62.23
Annotated in NT	30,702	36.35
Annotated in KEGG	21,930	25.96
Annotated in Swiss-Prot	39,135	46.34
Annotated in PFAM	39,660	46.96
Annotated in GO	39,659	46.96
Annotated in KOG	18,134	21.47
Annotated in all Databases	7945	9.40
Annotated in at least one Database	60,337	71.44
Total unigenes	84,450	100

**Table 3:** The 10 metabolic pathways with the largest number of unigenes.

Pathway Hierarchy	KEGG Pathway	Pathway ID	Gene Number
Genetic Information Processing-Translation	Ribosome	ko03010	1236
Metabolism-Global and overview maps	Carbon metabolism	ko01200	865
Metabolism-Global and overview maps	Biosynthesis of amino acids	ko01230	790
Genetic Information Processing-Folding, sorting and degradation	Protein processing in endoplasmic reticulum	ko04141	677
Genetic Information Processing-Transcription	Spliceosome	ko03040	675
Genetic Information Processing-Translation	RNA transport	ko03013	571
Cellular Processes-Transport and catabolism	Endocytosis	ko04144	508
Metabolism-Nucleotide metabolism	Purine metabolism	ko00230	499
Metabolism-Energy metabolism	Oxidative phosphorylation	ko00190	481
Genetic Information Processing-Folding, sorting and degradation	Ubiquitin mediated proteolysis	ko04120	396

**Table 4:** Dormancy release genes of *N. roborowskii* seeds.

ID	Description	Count	Up	Down	Padj
ko04075	Plant hormone signal transduction	146	126	20	6.79 × 10^−15^
ko00940	Phenylpropanoid biosynthesis	116	110	6	1.35 × 10^−9^
ko00941	Flavonoid biosynthesis	27	27	0	0.00262
ko00500	Starch and sucrose metabolism	122	108	14	0.003157
ko00591	Linoleic acid metabolism	20	20	0	0.008
ko00360	Phenylalanine metabolism	63	56	7	0.011109
ko05140	Leishmaniasis	48	44	4	0.014023
ko00073	Cutin, suberin and wax biosynthesis	26	23	3	0.023415
ko04722	Neurotrophin signaling pathway	77	70	7	0.0457
ko04620	Toll-like receptor signaling pathway	50	46	4	0.047342

**Table 5:** Hormone metabolism-related differentially expressed genes.

Biological Process	Unigenes ID	FPKM Value of Gene		Annotation
		Dormant Seeds	Dormant Release Seeds	
GA	Cluster-27177.30145	2.01	19.48	Probable monogalactosyldiacylglycerol synthase, chloroplastic
	Cluster-27177.18711	41.22	337.4	longation factor Tu, chloroplastic
	Cluster-27177.19727	2.91	21.23	Glycine-tRNA ligase, chloroplastic/mitochondrial 2
	Cluster-27177.13544	8.21	54.98	Desiccation protectant protein Lea14 homolog
	Cluster-27177.1710	1.55	10.97	Cytochrome P450 71D9
	Cluster-27177.34044	25.88	138.5	14-3-3-like protein D
	Cluster-27177.29822	5.32	29.52	Biotin carboxyl carrier protein of acetyl-CoA carboxylase, chloroplastic
	Cluster-27177.35850	8.48	43.6	Sulfite reductase [ferredoxin], chloroplastic (Fragment)
	Cluster-27177.22067	59.16	263.84	Profilin-2
	Cluster-27177.34997	6.54	25.56	Glycine-rich domain-containing protein 1
	Cluster-27177.15086	3.08	9.92	(S)-ureidoglycine aminohydrolase
	Cluster-27177.13522	1.41	15.89	DELLA protein GAI
	Cluster-27177.27204	5.56	41.42	DELLA protein GAI
	Cluster-27177.21248	16.13	50.46	DELLA protein GAIP
	Cluster-27177.26304	68.8	11.26	Zinc finger protein GAI-ASSOCIATED FACTOR 1
ABA	Cluster-27177.2324	36.05	23.32	Abscisic acid receptor PYL4
	Cluster-27177.12799	3.15	36.5	Abscisic acid receptor PYL4
	Cluster-27177.43406	5.24	33.02	Abscisic acid receptor PYL1
	Cluster-27177.25231	72.89	11.98	Abscisic acid receptor PYL3
	Cluster-27177.23379	193.62	0.99	Abscisic acid receptor PYL12
IAA	Cluster-27177.10496	0	18.13	Auxin transporter-like protein 3
	Cluster-27177.16821	0.41	60.75	Auxin transporter-like protein 2
	Cluster-27177.27836	1.59	17.72	Protein TRANSPORT INHIBITOR RESPONSE 1
	Cluster-27177.24531	56.87	35.43	Protein TRANSPORT INHIBITOR RESPONSE 1
CTK	Cluster-27177.36254	0.06	11.86	Histidine kinase 1
	Cluster-27177.13653	0.1	7.88	Histidine kinase 4
	Cluster-27177.33706	0.82	21.81	Histidine kinase 3
ETH	Cluster-27177.33263	1.84	44.82	Probable ethylene response sensor 1
	Cluster-27177.19483	4.78	12.75	Ethylene receptor
	Cluster-27177.34307	1.46	11.33	Ethylene receptor 2
BR	Cluster-27177.15305	7.81	33.97	Somatic embryogenesis receptor kinase 1
	Cluster-27177.8489	5.25	21.89	Brassinosteroid-responsive RING protein 1
	Cluster-27177.35137	0.39	37.81	Transcription factor TGA7
	Cluster-27177.34965	2.69	21.51	Transcription factor TGA9
	Cluster-27177.20740	3.57	13.29	Transcription factor TGA4
	Cluster-27177.36872	0	235.47	Pathogenesis-related protein 1
	Cluster-27177.16764	0	254.94	Pathogenesis-related protein 1
JA	Cluster-14375.0	0	2.78	Secretory phospholipase A2 receptor
	Cluster-27177.32969	0.64	193.54	Alpha-dioxygenase 1
	Cluster-27177.41108	0.03	8.95	Alpha-dioxygenase 2
	Cluster-27177.41251	0	3.2	Alpha-dioxygenase 1
	Cluster-27177.944	0	2.23	Jasmonate O-methyltransferase

Notes: GA, gibberellin; ABA, abscisic acid; IAA, auxin; CTK, cytokinin; ETH, ethylene; BR, brassinolide; JA, jasmonic acid.

## Data Availability

All authors warrant that their manuscript complies with ethical standards and meets the industry-recognized benchmarks reflected in MDPI’s policies.
